# Cross-lagged relationship between anxiety, depression, and sleep disturbance among college students during and after collective isolation

**DOI:** 10.3389/fpubh.2022.1038862

**Published:** 2022-12-06

**Authors:** Congying Shi, Shujian Wang, Qihui Tang, Xiangping Liu, Yue Li

**Affiliations:** ^1^School of Psychology, Nanjing Normal University, Nanjing, China; ^2^Faculty of Psychology, Beijing Normal University, Beijing, China; ^3^Beijing Key Laboratory of Applied Experimental Psychology, National Demonstration Center for Experimental Psychology Education, Beijing, China; ^4^Faculty of Arts, Shenzhen University, Shenzhen, China

**Keywords:** anxiety, depression, sleep disturbance, cross-lagged panel model, college students

## Abstract

**Object:**

Repeated quarantine policies over the past 3 years have led to poor psychological consequences for the public. Previous studies have proved that the quarantine policy leaves individuals vulnerable to anxiety, depression, and insomnia, especially among college students. This study aims to explore whether psychological problems during isolation continue with the release of isolation.

**Methods:**

Overall, 2,787 college students both answered a web-based survey during and after the closure management was lifted. The Patient Health Questionnaire, Generalized Anxiety Disorder Scale, and Youth Self-rating Insomnia Scale were measured. The cross-lagged path model was used to explore the influence of psychological impact during isolation on the individual after the release.

**Results:**

We found that anxiety and sleep disturbance levels alleviated significantly after quarantine, except for depression. As expected, a bidirectional relationship exists between anxiety, depression, and sleep disturbance. Moreover, depression and sleep disturbance can predict post quarantine depression, sleep disturbance, and anxiety, yet anxiety cannot predict sleep disturbance afterward.

**Conclusion:**

Timely and effective intervention for anxiety, depression, and insomnia during isolation is essential for individuals to repair themselves quickly after the release.

## Introduction

At the end of 2019, Coronavirus disease was reported in China. COVID-19 spread quickly throughout the world to its highly contagious property, and the World Health Organization (WHO) declared it a global pandemic (1). Containment measures, including social distance, quarantine, or even city lockdown, are effective in disease containment, whereas stringent measures have introduced a greater risk of mental illness to the public (2). Evidence shows that the prevalence of mental illness during the pandemic was higher than in pre-pandemic (2–4). For college students, the impact of the pandemic on mental and physical health was worse (3, 5). Among mental health problems, depression and anxiety are the most prevalent in college students (6), negatively affecting mental health and daily performance (7). Meanwhile, data from several studies also suggest that sleep disturbance is prevalent in college students, impairing daytime function (5, 8). Previous research has established that anxiety, depression, and sleep disturbance are intertwined (8, 9). However, due to different measurements, no longitudinal studies, whether the relationship between anxiety, depression, and sleep disturbance alters with time is not yet clear (3, 10), as if the interpretation of this relationship will benefit the prevention and intervention of anxiety, depression, and sleep disturbance after the pandemic. Given this, our study aims to reveal the relationship between anxiety, depression, and sleep disturbance.

In terms of the relationship between anxiety and depression, previous studies established that depression and anxiety are two quotidian comorbidities (8, 11, 12). However, a much-debated question is whether the longitudinal relationship between anxiety and depression is bidirectional. Several studies demonstrate that anxiety precedes the advent of depression (12–14). Different from these studies, other studies suggest that depression precedes the advent of anxiety (15, 16). Apart from the statement that the relationship between anxiety and depression is unidirectional, a meta-analysis focusing on longitudinal studies demonstrates that the relationship between anxiety and depression is bidirectional (11). However, with a sample from western countries, we cannot establish a bidirectional relationship between anxiety and depression in Chinese samples (11). Considering the controversy of the relationship between anxiety and depression, further research about this will be needed, and we put forward the following hypothesis:

Hypothesis 1. There would be a bidirectional relationship between anxiety and depression during the pandemic.

There are mainly three different viewpoints regarding the relationship between depression, anxiety, and sleep disturbance. According to cognitive models, the first viewpoint is that depression and anxiety are predictors of sleep disturbance, for fear, worry, and anxiety can cause insomnia (17). However, Buysse et al. (18) asserted that sleep disturbance is the prefigure of depression through a longitudinal cohort study. Furthermore, several review articles show that sleep disturbance is a risk factor for both anxiety and depression (7, 19, 20). Inspired by two unidirectional viewpoints, recent evidence suggests that the relationship between depression, anxiety, and sleep disturbance is bidirectional instead. On the one hand, depression and anxiety will lead to sleep disturbance. And conversely, suffering from sleep disturbance will result in the occurrence or development of anxiety and depression. Cui et al. (3) has done a cross-sectional survey in China, suggesting that the relationship between anxiety, depression, and sleep disturbance is bidirectional. The result of this study is consistent with several other studies (21–23). However, to our knowledge, many studies that suggest bidirectional relationships are cross-sectional or meta-analysis studies. Little longitudinal studies determine the directionality and causality between anxiety, depression, and sleep disturbance (3, 8). Although debate continues, given the situation that the last viewpoint is the combination of the first two ideas, we pose the following hypothesis:

Hypothesis 2. There would be a bidirectional relationship between sleep disturbance and depression.
Hypothesis 3. There would be a bidirectional relationship between sleep disturbance and anxiety.

In addition, previous research established that gender may influence the relationship between anxiety, depression, and sleep disturbance. First of all, as for the prevalence of anxiety, depression, and sleep disturbance, evidence shows that there is a discrepancy between males and females. Using meta-analysis, a study collected and analyzed 98 previous studies, suggesting that female students had a higher prevalence of depression and anxiety than male students (2). In terms of the prevalence of sleep disturbance, large-scale research showed that females had higher total Pittsburgh Sleep Quality Index (PSQI) scores than males, indicating that females had more sleep problems (8). Consistent with this result, other studies demonstrated that females were prone to symptoms of anxiety, depression (24, 25), and sleep disturbance (26, 27). Conversely, some studies suggest that the incidence of anxiety, depression, and sleep disturbance is higher in males than females (28), even not significant (29). However, as for the question of whether gender affects the relationship between anxiety, depression, and sleep disturbance, few studies have investigated it (30, 31), and further study is worthy. Considering the controversy and paucity of this area, we put up the following hypothesis:

Hypothesis 4. There would be a gender difference in the relationship between anxiety, depression, and sleep disturbance.

The current longitudinal study collected data from Chinese college students, conducting cross-lagged analysis to examine the relationship between anxiety, depression, and sleep disturbance. Additionally, this study examined the gender difference in the relationship between anxiety, depression, and sleep disturbance through multiple-group analysis. The results of this study will provide further evidence of the relationship between anxiety, depression, and sleep disturbance. Moreover, given that COVID-19 worsens people's mental and physical health, the results of this study will also provide information on the prevention and intervention of anxiety, depression, and sleep disturbance.

## Methods

### Participants

The first wave of data was obtained from 6,710 college students during the COVID-19 pandemic lockdown in Harbin on September 26, 2021. The second wave of data was collected from 3,731 college students from the same school after the closure management was lifted on December 27, 2021. When the datasets from two waves were combined according to the students' school numbers, 2,787 participants (58.6% females, *Mean*_age_ = 18.34, *SD*_age_ = 0.92, range from 15 to 28) were finally recruited. Students and their parents had to provide signed informed consent before participating in the assessment. All participants answered the questionnaires through the Wenjuanxing online questionnaire platform (https://www.wjx.cn/). The research was examined and approved by the ethical committee of Beijing Normal University (Reference number: 202112220084).

### Measures

#### Patient health questionnaire (PHQ-9)

The Patient Health Questionnaire (PHQ-9) is a widely used scale for screening depression symptoms (32). Participants were asked about the frequency [not at all (0), several days (1), more than half of the days (2), nearly every day (3)] of experiencing given symptoms in the last 2 weeks, and higher scores indicate more severe depression symptoms. The Chinese version of PHQ-9 was proved valid and reliable (33). In the current study, PHQ-9 has a high internal consistency with Cronbach α values of 0.89 and 0.92 in wave 1 and wave 2, respectively.

#### Generalized anxiety disorder scale (GAD-7)

The Generalized Anxiety Disorder Scale (GAD-2) is a valid and reliable assessment to screen for generalized anxiety symptoms (34). Participants answered seven questions about the frequency of anxiety symptoms that occurred over the last 2 weeks. Each item scored from 0 (not at all) to 3 (nearly every day), with a higher score indicating more severe anxiety symptoms. The Chinese version also has good psychometric properties for identifying anxiety (35). In the current study, GAD-7 has a high internal consistency with Cronbach α values of 0.93 and 0.95 in wave 1 and wave 2, respectively.

#### Youth self-rating insomnia scale (YSIS-8)

The Youth Self-rating Insomnia Scale (YSIS-8) is a 5-point Likert questionnaire assessing sleep disturbance in the last month. Participants answered two questions about overall sleep quality and six about the frequency of specific sleep disturbance symptoms. Total scores range from 3 to 15, and higher scores indicate poorer sleep quality. Previous studies have shown that YSIS-8 in Chinese is valid and reliable (36). In the current study, YSIS-8 has a high internal consistency with Cronbach α values of 0.91 and 0.93 in wave 1 and wave 2, respectively.

### Data analyses

Preliminary analyses were conducted in SPSS 22.0. The cross-lagged paths and multiple-group analysis were performed *via* Mplus 8.3. Because depression and anxiety symptoms in adolescents and young adults change over time (37), age was included as a covariate in the model. It should be mentioned that all variables were assumed to be related to all others; therefore, this model had zero degrees of freedom, and it did not make sense to assess model fit (38) except for multiple-group analysis.

## Results

### Preliminary analyses

Means, standard deviations, correlations, and *t*-test results of major variables are shown in Table 1. The results demonstrated that anxiety, depression, and sleep disturbances were significantly and positively related to each other at each wave. Additionally, after 3 months when the lockdown ended, there was no significant change in the level of depression. At the same time, the level of anxiety and sleep disturbances have decreased significantly.

**Table 1:** Preliminary analysis for major variables (*n* = 2,787).

**Variables**	**M**	**SD**	**1**	**2**	**3**	**4**	**5**	***t***	***p***
1. Wave 1 depression	2.79	3.91						−0.92	0.35
2. Wave 2 depression	2.86	4.44	0.45^**^						
3. Wave 1 anxiety	1.83	3.18	0.82^**^	0.42^**^				3.80	<0.01
4. Wave2 anxiety	1.58	3.17	0.40^**^	0.78^**^	0.42^**^				
5. Wave 1 sleep	12.3	5.29	0.64^**^	0.39^**^	0.60^**^	0.31^**^		2.13	0.03
6. Wave 2 sleep	12.1	5.60	0.37^**^	0.65^**^	0.35^**^	0.64^**^	0.45^**^		

** *p* < 0.01.

### The cross-lagged path model

**Figure 1:**
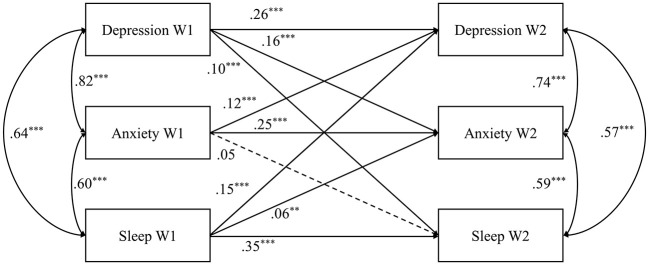
The standardized cross-lagged model between depression, anxiety, and sleep disturbances. The residuals were not shown; **p < 0.01, ***p < 0.001.

The cross-lagged regression model was performed to analyze the causal link between anxiety, depression, and sleep disturbances while controlling by age. As shown in Figure 1, all three constructs demonstrated strong stability over 3 months, with autoregression path coefficients ranging from 0.25 to 0.35. Moreover, after controlling the autoregression of anxiety, depression, and sleep disturbances, as well as the correlation of three constructs at the same wave, depression at wave 1 could significantly predict anxiety (*β* = 0.16, *p* < 0.001) and sleep disturbances (*β* = 0.10, *p* < 0.001) at wave 2. Anxiety at wave 1 could significantly predict depression (*β* = 0.12, *p* < 0.001) at wave 2. Sleep disturbances could significantly predict depression (*β* = 0.15, *p* < 0.001) and anxiety (*β* = 0.06, *p* < 0.01) at wave 2. However, anxiety at wave 1 could not significantly predict sleep disturbances (*β* = 0.05, *p* = 0.08). These results indicated that the relationship between anxiety and depression, sleep disturbances, and depression is bidirectional, while the relationship between anxiety and sleep disturbances is unidirectional.

A multi-group analysis grouped by gender was conducted to examine gender differences in the cross-lagged model. We constructed a structural weights equivalent model, supposing that regression coefficients were equal between genders. Fitting results were excellent referencing to Hu and Bentler's criteria (39): χ^2^ = 15.99, *df* = 9, *CFI* = 0.99, *TLI* = 0.99, *RMSEA* = 0.02 (90% *CI* = [0.00, 0.04]). We compared the structural weights equivalent model to the unconstrained model to test whether there is a gender difference. The results showed that Δχ^2^ = 15.99, Δ*df* = 9, *p* > 0.05) indicated no significant gender difference in the cross-lagged model of depression, anxiety, and sleep disturbances among Chinese college students (see Table 2).

**Table 2:** Pairwise parameter comparisons.

	**Male**		**Female**	
**Path**	***β***	***p***	***β***	***p***
Wave 1 depression → Wave 2 depression	0.19	<0.01	0.32	<0.01
Wave 1 depression → Wave 2 anxiety	0.12	<0.01	0.19	<0.01
Wave 1 depression → Wave 2 sleep	0.12	=0.02	0.08	=0.03
Wave 1 anxiety → Wave 2 depression	0.16	<0.01	0.09	=0.01
Wave 1 anxiety → Wave 2 anxiety	0.28	<0.01	0.22	<0.01
Wave 1 anxiety → Wave 2 sleep	0.08	=0.10	0.03	=0.35
Wave 1 sleep → Wave 2 depression	0.12	<0.01	0.18	<0.01
Wave 1 sleep → Wave 2 anxiety	0.02	=0.53	0.09	<0.01
Wave 1 sleep → Wave 2 sleep	0.26	<0.01	0.42	<0.01

## Discussion

Studies on sleep disturbance, depression, and anxiety have lasted for over 40 years (40). However, with the foreground of the pandemic of COVID-19, depression, anxiety, and sleep disturbance have become more prevalent, especially among college students. Lack of longitudinal studies, our results portrayed several findings as follows. Some points are worth discussing.

Inconsistent with the result of Wang et al. (41), which indicated that anxiety levels of high school students in Wuhan raised with the new semester after being quarantined for several months in the first wave of the pandemic. Our result provides sequential proof that after home quarantine, anxiety alleviated significantly in the situation that the pandemic has already lasted for over 1 year. Our results also may support the hypothesis that increased physical activities can mitigate the anxiety level of college students (42), for the anxiety level is reduced after quarantine which means more space and opportunities for physical activities can buffer anxiety. Even if no previous studies revealed the fluctuation of sleep disturbance, after quarantine, sleep quality also resurrects to some degree. Combined with previous studies, besides physical activities, augmentation, improvement, or a stable diet may contribute to sleep disturbance resurrection (43).

In contrast, as if levels of depression seemed to increase with time, the comparison is not significant on a statistical level. To deduce why college students did not get rid of the shackle of depression after home quarantine, clues from Xiang et al. (42) implied that only when physical activities reach a moderate level can depression be mitigated. Therefore, in the situation that college students did not do enough physical activities, depression was not meliorated at an effective level.

Referring to associations between anxiety, depression, and sleep disturbance, only could anxiety just predict depression and anxiety afterward, while depression and sleep disturbance can simultaneously predict anxiety, sleep disturbance, and depression. However, previous studies portrayed a bidirectional relationship between anxiety and sleep disturbance, which indicated that anxiety and sleep disturbance could predict mutually (44, 45). However, from our results, the situation varies with the foreground of COVID-19, a pandemic. Tao et al. (46) researched symptoms of sleep disturbance, anxiety, and depression, a change in sleep disturbance can change the anxiety and depression structure. Hence, it is proper to speculate that before anxiety is alleviated after quarantine, sleep quality improves in advance. Therefore, from this point of view, anxiety cannot predict sleep disturbance.

Besides disease association, though no studies contain a comparison between pre-pandemic, during the pandemic, and post-pandemic, we can draw some clues from previous studies to raise surmises and supply information to patch a holistic view. From the perspective of cross-sectional data, correlations exist between anxiety, depression, and sleep disturbance which is coherent with the results of Becker et al. (8). From data of wave 1 and wave 2, there is a significant bidirectional relationship between anxiety, depression, and sleep disturbance. When we make the comparison between wave 1 and wave 2, the relationship between anxiety, depression, and sleep disturbance becomes less close. Though no studies were done during the pandemic and post the pandemic, research done by Deng et al. (2) showed that levels of anxiety, depression, and sleep disturbance reached the peak level during the pandemic. Our results provide information from another facet after quarantine, the relationship between anxiety, depression, and sleep disturbance becomes sparser. In the domain of hopelessness, a factor related to anxiety and depression, Tao et al. (47) drew the same trend in college students, which implied that after quarantine, hopelessness was reduced. Combining previous studies' results, we estimate that less time spent on mobile phones and more physical activities can reduce anxiety, depression, and sleep disturbance and further dilute the bidirectional relationship (42, 48).

## Limitation

First, as discussed above, the severity of depression, anxiety, and sleep disturbance can be affected by many externals, such as school schedule and containment measurement. Some other information, such as time of sleep and frequency of physical activities can be confounding factors influencing degrees of anxiety, depression, and sleep disturbance. Second, anxiety, depression, and sleep disturbance can alter with quarantine duration and time after quarantine duration. Hence, in future studies, more time points, time duration, and other pandemic containment measures can be pinned for investigation.

## Conclusion

In our current study, three main findings were (1) levels of anxiety and sleep disturbance alleviated significantly after quarantine except for depression; (2) a bidirectional relationship exists between anxiety, depression, and sleep disturbance; (3) depression and sleep disturbance can predict post quarantine depression, sleep disturbance and anxiety, yet anxiety cannot predict sleep disturbance afterward. Our current study clearly shows that quarantine adds great pressure and stress on college students while, after quarantine, college students' mental status resumes great resilience.

## Data availability statement

The raw data supporting the conclusions of this article will be made available by the authors, without undue reservation.

## Ethics statement

The studies involving human participants were reviewed and approved by Beijing Normal University. The patients/participants provided their written informed consent to participate in this study.

## Author contributions

CS took the lead in writing the manuscript. YL conceived the study design and supervised the data collection. SW performed the data analysis. QT, XL, and YL provided critical feedback and helped shape the research, analysis, and manuscript. All authors contributed to the article and approved the submitted version.

## Conflict of interest

The authors declare that the research was conducted in the absence of any commercial or financial relationships that could be construed as a potential conflict of interest.

## Publisher's note

All claims expressed in this article are solely those of the authors and do not necessarily represent those of their affiliated organizations, or those of the publisher, the editors and the reviewers. Any product that may be evaluated in this article, or claim that may be made by its manufacturer, is not guaranteed or endorsed by the publisher.
